# Supplementary material for: Cross sectional analysis of clinical trials search results for cancer patients using a navigator-assisted clinical trials search using five different search engines
Source: PLoS One. 2025 Jun 25;20(6):e0326139. doi: 10.1371/journal.pone.0326139 (PMC12193830; doi:10.1371/journal.pone.0326139)
Supplement: Attachment — Submitted filename: Response to Reviewers.docx [file pone.0326139.s004.docx]

**PlosONE Reviewer Comments**

BLACK = reviewer’s commentary.

RED = Examples of modifications in the paper related to the comment.

GREEN = Reply back to the comment

5. Review Comments to the Author

Please use the space provided to explain your answers to the questions above. You may also include additional comments for the author, including concerns about dual publication, research ethics, or publication ethics. (Please upload your review as an attachment if it exceeds 20,000 characters)

**Reviewer #1:**

1. The criteria for not considering more clinical trial searchers are not very clear, which could provide more information to analyze the possible deficiencies that each one presents when searching for clinical trials. Similarly, the criteria for not considering a longer study time.
   a. Reply back: See below manuscript additions.
   b. Line 136: We opted not to extend the study duration beyond September due to the implementation of new clinical trial navigators at the conclusion of this timeframe. This decision was made to maintain consistency in participant interaction and data collection methodologies throughout the study. In addition, having roughly received 100 patients referrals a year, the 18 patients were considered a reasonable proportion of the yearly total.
   c. Line 152: The number of clinical trial navigators was limited to three as at the time of the research project, we only had three trained navigators to perform the clinical trial searches..
2. There is no flowchart or graphic diagram detailing the procedure or standardized methods used to analyze the results of the search for clinical trials for cancer patients.
   a. Reply back: See below manuscript additions.
   b. Line 224: Fig 1 summarizes the procedure followed during the study period for data collection by each navigator.
   c. **Fig 1. Graphical representation of the methodologies**.

d. CT.gov = ClinicalTrials.gov. *Undiscoverable trials = trials not found on the initial ClinicalTrials.gov search but found on alternative website and present on the Clinicaltrials.gov website.

3. A percentage representation of the problems encountered in the categorized search engines (lack of reproducibility, lack of searchable trials, lack of standardized and inclusive search terms, errors in the search engines, and inadequate or outdated information to complete a meaningful trial search) and a representation of the relationship between these problems and the search engines are not shown. I recommend incorporating more representations of statistical analysis.
   a. Reply back: We were able to complete an additional representation statistical analysis comparing the proportion of eligible trials found on ClinicalTrials.gov versus alternative websites.
      i. *Line 248: “To compare the proportion of trials identified on ClinicalTrials.gov versus alternative website, a one-sample proportion test was conducted.”*
      ii. *Line 371: “The proportion of trials identified from ClinicalTrials.gov was less than 50% (p = 0.021)”*
   b. We are unable to provide percentage representation for all of the problems encountered in the categorized search engines because of the way the study was done: as a discovery/grounded theory analysis. At this point, we were identifying the themes of the challenges rather than the percentages of the problems. We do have statistics on certain challenges, such as the lack of co-location and cohort status as this is present 100% of the time. We have added commentary in the paper’s discussion about this and how it presents a potential area for future research. Subsequent investigations will elucidate the percentages of problems on these websites. In addition, a representation of the relationship between these challenges and the search engines was discussed. For example, we talk about specific examples tied to certain websites, and we mention the specific websites that displayed a lack of reproducibility:
      i. *Line 302: Our study identified a lack of reproducibility of search results on ClinicalTrials.gov, Canadian Clinical Trials and Ontario Clinical Trials.*
   c. Line 245: “To compare the proportion of trials identified on ClinicalTrials.gov versus alternative website, a one-sample proportion test was conducted.”
   d. Line 369: “The proportion of trials identified from ClinicalTrials.gov was less than 50% (p = 0.021)”
   e. Line 398: “The websites never provided important details such as which cohort of the study is currently open” perhaps change this to: “ No website provided up-to-date information such as which cohort of the trials was open, and whether the clinical trial site had an open spot for patients.
   f. Line 407: “Our study identified a complete lack of co-location filtering in ClinicalTrials.gov, 3CTN, and Canadian Cancer Trials” (you will need to explain this… )
   g. Line 435: “Due to the scoping nature of our qualitative analysis, we did not assess the relative representation of these challenges within the websites. Certain errors, such as lack of co-location and information on cohort status, were consistently identified. Further investigation into the frequency of categorized search engine problems (lack of reproducibility, undiscoverable trials, lack of standardized search terms, inadequate or outdated information) will be pursued in a future study..”
4. I think you should incorporate other current studies so that you can incorporate them in the discussion of results, as well as other recommendations (based on the new incorporation) to improve the functionality of the search engines for clinical trials.
   a. Reply back: We have expanded our discussion to include studies that support our findings, provide additional examples, and additional barriers. In addition, we have expanded the discussion with potential opportunities to improve the current status in challenges of clinical trials searches. Lastly, we have included studies that support the use of a navigator-assisted clinical trial search program as a potential solution.
   b. Line 600: We have developed Master Lists for each large disease site that are maintained and kept up to date. We review five clinical trial search engines routinely to ensure a comprehensive list of clinical trials is created. These are used for clinical trials searches and found to be more reliable than repeating searches on clinical trials sites

I consider that 23 bibliographic sources are too few. Perform a literature search and add results, experiences, and current trends to the search for clinical trials for cancer patients. More than 65% of the references are more than 5 years old.

a. Reply back: We were able to identify 13 additional references. In addition, we performed a scoping review using key terms to ensure that we were including all relevant literature in our paper. Please note that we were experiencing errors with Zotero, the citation manager, when trying to track changes on Word, so the citation edits were made in the “Editing” Word view. We apologize for this. The rest of the edits were made with track changes in the manuscript document that has the track changes.

**Reviewer #2**: This paper presents a study that addresses the limitations in clinical trial search engines by conducting a critical analysis of several prominent websites, including [ClinicalTrials.gov](http://ClinicalTrials.gov), Canadian Cancer Trials, Clinical Trials Ontario, Canadian Cancer Clinical Trials Network, and Q-CROC. The study underscores the need for improvement in the current clinical trial search system to enhance patient outcomes. The authors discuss challenges, and their findings can potentially serve as a foundation for developing enhanced search engines with improved functionality. This advancement would enable healthcare professionals and patients to find and access the most suitable clinical trials with greater ease and accuracy. However, upon careful consideration, it appears that the content and scope of this paper may not align with the expected caliber or scholarly depth typically associated with the targeted journal for publication.

Reply back: We appreciate the commentary. We have expanded our discussion after a scoping review of the literature to improve the scholarly depth of our paper. We have also enhanced our statistical analysis to improve reproducibility and have been able to offer concrete suggestions that future researchers will be able to use to improve the current status of clinical trials searches. We believe that these changes offer an adequate improvement in the scholarly depth of this manuscript.

**Reviewer #3**: The authors have investigated the challenges in finding appropriate clinical trials for Canadian patient suffering from cancer using [ClinicalTrials.gov](http://ClinicalTrials.gov) as well as 4 other websites (Canadian Cancer Trials, Clinical Trials Ontario, Canadian Cancer Clinical Trials Network, and Q-CROC) by conducting searches for 18 patients by three trained clinical trial navigators. They identified important challenges such as lack of reproducibility, out-of-date information, undiscoverable trials, lack of standardized and inclusive search terms, and search engine failures.

Overall, this is a well-designed study addressing the important issue of reliability of these clinical trial search engines in identifying all eligible trials for patients diagnosed with cancer. Also, the manuscript is easy to read and follow. Even though this is a great effort, some questions and concerns need to be addressed:

1. The authors have assigned different alternative websites to different trial navigators. Given the relatively large variability in the number of trials found in [ClinicalTrials.gov](http://ClinicalTrials.gov) by different navigators, it seems beneficial to repeat the search of alternative website by other navigators to check the variability of findings in these websites as well. What is the reason that the search in alternative websites were not done by all navigators? Do you expect it to change the findings? If not, it needs to be discussed in the manuscript.

Reply back: Our goal was to examine ClinicalTrials.gov in light of the other search engines, and not necessarily focus on the alternative websites. Thus, although interesting, this was not the scope of the study and would not change the findings of the study as there would still be a presence of undiscoverable trials on ClinicalTrials.gov regardless of if the alternative websites were searched by one navigator or all three.

2. As mentioned before, it is easy to follow and read the manuscript, however it seems redundant in some parts. For example, the time interval of the study has been mentioned with details on few occasions. It can only be mentioned in the Method section or Study Design section. Removing redundant sentences in the manuscript can improve the quality of the manuscript and keep the readers engaged.

Reply back: Thank you for the feedback. We have gone through and removed redundant sentences.

3. What are some of the changes that can potentially improve the challenges identified in this study. It worth to discuss some of these solutions in the Discussion section.

Reply back: We have included a discussion on potential solutions to the challenges we have identified in the study.

4. One of factors affecting the search outcome is the information listed in the referral forms. What information can be added to the referral forms to help with the challenges you have identified, if any? Is the level of variability in number of trials found by the navigators for each patient related to the extent of the info provided in the referral forms?

Reply back: Our navigators did not note any of the challenges being related to the lack of information on the referral forms. However, one piece of information that can be improved upon to the forms is biomarker status. Due to the diversity in biomarkers, patient biomarker status is often incomplete, and this can affect one’s ability to do an adequate search. We are addressing this through a separate study.

Reply back: We believe that the level of variability in number of trials found by the navigators for each patient was not related to the to the extent of information in the referral form. Because the navigators had access to the same information via a standardized referral form, all searches were done using the same patient information; thus, we don’t think it affected our results.

Challenges in improving intake sheets for the Clinical Trials Navigator are ongoing. We strive to balance the needs for extensive information needed for a comprehensive and accurate search, with the challenges of the patient knowledge of the level of detail needed for this type of search. We have a developed an improved process at the host site of the CTN which is rolling out to our expansion sites. We are embedding research associates in each of the cancer sites that will be able to gather personal health information needed for comprehensive searches and enter this data into the CTN website.

The sample size was too small to identify an improved outcome for patients from the host site and patients from outside the host site, to indicate if the challenges of the clinical trials search engine were not related to lack of information available for the clinical trials searches.

5. In line 112, the comma after “Functional” should be changed to “and”.

Reply back: the change was made.

6. In line 293: “Out of 54 [ClinicalTrials.gov](http://ClinicalTrials.gov) searches, only 5 searches revealed identical clinical trials results between navigators and four of these were when zero trials were found”, 54 should be changed to 18.

Reply back: To clarify, we put 54 instead of 18 because in total 18 patients x 3 navigators led to 54 searches conducted on ClinicalTrials.gov. We have modified the sentence to make it clearer.

6. PLOS authors have the option to publish the peer review history of their article ([what does this mean?](https://journals.plos.org/plosone/s/editorial-and-peer-review-process#loc-peer-review-history)). If published, this will include your full peer review and any attached files.


If you choose “no”, your identity will remain anonymous but your review may still be made public.
