# Supplementary material for: Cross sectional analysis of clinical trials search results for cancer patients using a navigator-assisted clinical trials search using five different search engines
Source: PLoS One. 2025 Jun 25;20(6):e0326139. doi: 10.1371/journal.pone.0326139 (PMC12193830; doi:10.1371/journal.pone.0326139)
Supplement: Attachment — Submitted filename: Reply to Reviewers Comments.docx [file pone.0326139.s005.docx]

**PlosONE Reply to Reviewer Comments**

BLACK = reviewer’s commentary.

RED = Modifications in the paper related to the comment.

GREEN = Reply back to the comment

We sincerely appreciate your thoughtful and constructive feedback. Your insights have helped us refine our analysis and improve the clarity of our findings. Below, we address each of your concerns and outline the corresponding revisions made to the manuscript.

**Reviewer #4**: I found this paper an interesting read overall, and it is of interest also to those who work on systematic reviews and meta-analysis. The design is sound. statistical analysis and reporting do not fully convince me.

The authors report an analysis on difference between navigators in number of trials found per patient on clinicaltrials.gov, which they found non significant. There seems to be a typo there as they say it's not significant and then they write P<0.05, but in general I think p-values should be reported fully unless they are <0.001.

Yet, in this case the problem I have with that analysis is that the small sample size, together with the apparent heteroskedasticity, weakens the results a lot. A quasipoisson or a zero-inflated poisson generalized linear model would probably work better than Kruskal Wallis and gain a bit of power, at least. In any case, it feels bizarre to read in the same paper that results were inconsistent between navigators while also reporting that they do not differ significantly. Perhaps the toolset is inadequate to communicate the point. The thing of the greatest interest in such an analysis is not whether there are differences between couples of raters, but a general measure of variability or agreement, which would make the use of the analysis of variance toolset very valuable here. By looking at the data structure, it seem that the very first data to report would be intraclass correlations for both "patient" and "navigator" as obtained from a random effect model. So, ideally, a poisson random effect model does the trick in the cleanest possible way, although a linear model would probably work too as a reasonable approximation. You cannot easily obtain p-values for random effects, but you usually don't need them as the ICCs paint a good picture already.

- We acknowledge the limitations of our initial analysis and have reanalyzed the data using a zero-inflated Poisson generalized linear model. This approach improves model fit and provides a more accurate representation of the variability in the number of trials found per patient.
- To better quantify agreement, we now report intraclass correlation coefficients (ICCs)
  - Navigator ICC: ~0%, indicating that navigator-related variability is negligible.
  - Patient ICC: 57.9%, suggesting that differences between patients contribute significantly to the variation in the number of trials found.
- Line 239-243: “To analyze the variability in the number of trials found on ClinicalTrials.gov during the initial search, a zero-inflated Poisson random effects model was performed with navigator and patient as random effects. To compare the proportion of trials identified on ClinicalTrials.gov versus alternative websites, the observed proportion with a 95% binomial confidence interval was reported.”
- Line 349-359: “A zero-inflated Poisson generalized linear mixed model with navigator and patient as random effects was fit the data to provide a more accurate representation of the variability in the number of trials found per patient. The intraclass correlation coefficients (ICCs) from navigator is around 0% indicating that navigator-related variability is negligible. While ICC from patients is 57.9%, suggesting that differences between patients contribute significantly to the variation in the number of trials found. These results highlight that inconsistencies in trial identification are primarily driven by patient-level factors rather than navigator-specific search behavior. This is an expected result, as each patient will have different demographics and eligibility.”

In general, I see this reporting of p-values even in instances where they don't really say anything relevant as the hypothesis itself is meaningless. How is it relevant to test the hypothesis that the number of trials found on other sites in less than 50% than what found on clinicaltrials.gov? It's just an arbitrary threshold, we could test if they were less than 75%, or 35%. In this case it is more sensible to just report the proportion with the binomial confidence interval. Plus, after spending so much energy to prove that the number of trials you find on clinicaltrials.gov is not consistent across searches, treating it as a fixed number as opposed to a random variable seems a contradiction: why should we consider it a gold standard, when it has been made clear that is so severely inconsistent?

- We acknowledge your concern regarding the arbitrary nature of testing. In response, we have removed hypothesis testing for this comparison and now report the observed proportion with a 95% binomial confidence interval instead. The proportion of trials found on other registries compared to ClinicalTrials.gov is 57% (95% CI: 50%, 63%). By presenting the confidence interval, we provide a more transparent and informative measure of the extent of trial identification differences across registries without relying on an arbitrary threshold.
- Line 45-46: added to ‘(57% “with 95% binomial confidence interval [50%, 63%]”)’
- Line 387-388: added to ‘(57% “with 95% binomial confidence interval [50%, 63%]”)’

The strange thing about this paper is that it is all about repeatability of results, and yet there is zero measures of repeatability or agreement, while there's plenty statistical tools made precisely for that purpose. It's perplexing and I think it should be re-worked.

- Your suggestion to include repeatability and agreement measures is well taken. We now incorporate: Intraclass correlation coefficients (ICCs) to assess navigator and patient variability. These additional measures provide a more robust assessment of search repeatability and reinforce our key findings.

As a side note, I would also add that in the particular field of research, i.e. literature searches, agreement should in theory pretty much be perfect, and even a simple description of results without any fancy statistics shows already that perfect it is not. I believe this is relevant already.

- Thank you for your time and expertise in reviewing this paper.

**Reviewer #5:** The revised manuscript titled "Cross-sectional analysis of clinical trials search results for cancer patients using a navigator-assisted clinical trials search using five different search engines" presents a thoughtful and detailed analysis of clinical trial search tools. This study addresses an important challenge: helping patients and healthcare providers find suitable clinical trials, which can often be life-changing. ClinicalTrials.gov, as one of the most widely used platforms, is highlighted as a crucial resource, but its limitations, along with those of other search tools, are well-documented in this work.

The manuscript is clear and well-organized, flowing naturally from the introduction to the conclusion. The authors have made noticeable improvements, particularly in detailing the methodology. They provide a deeper explanation of how patients were selected, how navigators were trained, and how the searches were conducted. These updates make the study more transparent and trustworthy. The added figure also help to visualize the comparisons and results more effectively.

One of the most valuable aspects of this study is its practical implications. By identifying issues such as inconsistent terminology, outdated information, and technical errors in current search tools, the authors propose clear, actionable solutions. These include expanding filters to include prior therapies, and improving user interfaces to make search engines more user-friendly. The idea of navigator-assisted searches is especially compelling, as it demonstrates a meaningful way to help patients gain better access to trials.

The authors have responded well to the reviewers’ initial comments, especially regarding inconsistencies in search results and differences between navigators.

However, I have a few additional suggestions:

1. the discussion should address why the analysis focuses on 2022 data when the manuscript was submitted in 2024. Readers might wonder about this gap, so providing a brief explanation would be helpful.

- *This study was originally submitted to PLOS One in May of 2023. We received an initial review with recommended revisions in March 2024, ten months later. We submitted our revisions in April 2024, and have been waiting for this review since then. Your review is now 11 months from the revision that was submitted in 2024. This explains the 2022 data.*
- Line 230: “Unavoidable delays were experienced in PLOS ONE delaying the publication of this paper, originally submitted in 2023”

2. the repeated mention of specific dates ("July 6, 2022, and September 14, 2022") throughout the manuscript should be streamlined to avoid redundancy, as suggested by reviewer #3.

- We have removed the exact dates from redundant places and replaced it with more concise verbiage.
- Line 106: replaced with “During the summer of 2022”
- Line 184: removed
- Line 285: replaced with “during the summer of 2022 accrual period”
- Line 295: removed

3. Since the study was conducted in 2022, the authors should acknowledge that search engines might have improved since then, which is an important limitation and should be mentioned in the conclusion.

- We added a section addressing this as a limitation in our discussion where we talk about study limitations.
- Line 587-591: “In addition, this study’s search engine analyses were conducted in 2022. Since then, these platforms may have undergone updates or improvements that could impact search accuracy, accessibility, or user experience. As a result, some of the challenges identified in this study may have been mitigated, while others may persist.”

In summary, this is a well-executed and highly relevant study that provides important insights into improving clinical trial search systems. With just a few small adjustments, it will be ready for publication. The findings have the potential to benefit not only patients but also healthcare providers and policymakers working to improve access to innovative treatments.

I am delighted to serve as a reviewer for this manuscript, as it significantly contributes to my clinical practice.

- Thank you for your time and expertise in reviewing this paper.
