# Supplementary material for: Cross sectional analysis of clinical trials search results for cancer patients using a navigator-assisted clinical trials search using five different search engines
Source: PLoS One. 2025 Jun 25;20(6):e0326139. doi: 10.1371/journal.pone.0326139 (PMC12193830; doi:10.1371/journal.pone.0326139)
Supplement: Attachment — Submitted filename: Response_to_Reviewers_auresp_3.docx [file pone.0326139.s006.docx]

PONE-D-23-23984R2

Cross-sectional analysis of clinical trials search results for cancer patients using a navigator-assisted clinical trials search using five different search engines

PLOS ONE

To Vahid Mansouri, M.D.
Academic Editor
PLOS ONE

 Thank you so much for your support on our paper:

1- We have double checked and the formatting of the references are aligned with the PLOS one referencing guideline.

2- *Please cite the conferences you have published a part of your work in. This has been included in the ‘Acknowledgment’ section.*

This manuscript has been partially presented in 2022 WE-SPARK Health Research Conference, 2023 UWill Discover Student Research Conference, and in abstract form for the American Society of Clinical Oncology, 2023.

3- The Manuscript Organization is fully aligned with the authors guideline (about naming of headings and supplementary materials).

Please find enclosed:

1. A rebuttal letter that responds to each point raised by the academic editor and reviewer(s). You should upload this letter as a separate file labeled **'Response to Reviewers'**. - submitted
2. A marked-up copy of your manuscript that highlights changes made to the original version. You should upload this as a separate file labeled **'Revised Manuscript with Track Changes'**. submitted
3. An unmarked version of your revised paper without tracked changes. You should upload this as a separate file labeled '**Manuscript**'. Submitted

Thank you for your time.

Caroline Hamm
